# A cognitive process occurring during sleep revealed by rapid eye movements

**DOI:** 10.1126/science.abp8852

**Published:** 2022-08-25

**Authors:** Yuta Senzai, Massimo Scanziani

**Affiliations:** 1 Department of Physiology, University of California San Francisco, San Francisco, CA, USA.; 2 Howard Hughes Medical Institute, University of California San Francisco, San Francisco, CA, USA.

## Abstract

Since the discovery of REM sleep, the nature of the rapid eye movements that characterize this sleep phase has remained elusive. Do they reveal gaze shifts in the virtual environment of dreams or simply reflect random brainstem activity? We harnessed the head direction (HD) system of the mouse thalamus, a neuronal population whose activity reports, in awake mice, their actual HD as they explore their environment and, in sleeping mice, their virtual HD. We discovered that the direction and amplitude of rapid eye movements during REM sleep reveal the direction and amplitude of the ongoing changes in virtual HD. Thus, rapid eye movements disclose gaze shifts in the virtual world of REM sleep, thereby providing a window in the cognitive processes of the sleeping brain.

## One-Sentence Summary

Rapid eye movements during REM sleep represent gaze shifts in the virtual world of the sleeping brain.

REM sleep is a phase of sleep characterized by rapid eye movements, from which it gets its acronym (1, 2). This phase of sleep is present across many vertebrates (3, 4) and has been associated with dreaming (5, 6). Indeed, when awakened during REM sleep, human subjects are more likely to report vivid dreams as compared to when awakened during other phases of sleep (7–10). This observation has led to the proposal that the nature of rapid eye movements during REM sleep may relate to the content of the ongoing dream (7–11). If so, rapid eye movements may represent a readout of some of the cognitive processes occurring in the sleeping brain. The verification of this hypothesis, however, has led to contradictory results. Some initial studies indicated a correlation between the content of dreams reported by the subject and the direction or frequency of rapid eye movements recorded immediately before awakening (7–10). However, other studies did not reproduce these results (12, 13). Furthermore, lifelong blind individuals who do not report visual experiences during dreams, do have rapid eye movements during REM sleep (14). Thus, alternative hypotheses suggested that rapid eye movements may be unrelated to the mental processes occurring during REM sleep and simply reflect random brainstem activity (11, 15).

Most of these studies, however, were based on the potentially inaccurate reporting of dreams by human subjects rather than on an objective measure of the cognitive processes occurring in the brain during REM sleep. Thus, we reasoned that by directly monitoring some of the cognitive processes occurring in the brain during REM sleep, we could gain insight as to whether rapid eye movements actually occur in coordination with such processes.

We decided to use the head direction (HD) system of the mouse as the objective readout. HD cells are a population of neurons present, among other structures, in anterodorsal nucleus of the thalamus (ADN). Their ensemble activity reports the direction of the head of the animal along the azimuth as it explores or navigates through its environment (16–18). During REM sleep, the population activity of HD cells is similar to that occurring during actual navigation (19, 20), thus potentially representing an internal “virtual heading” of the sleeping animal.

In awake, behaving mice exploring their environment, changes in head direction are accompanied by fast, saccade-like movements of the eyes in the same direction (21, 22). Does a similar coordination exist in the sleeping mouse? Clearly a sleeping mouse maintains a fixed heading. However, putative changes in the internal representation of heading of the sleeping mouse may be coordinated with the rapid eye movements occurring during REM sleep. By monitoring rapid eye movements we may be able to reveal changes in internal heading occurring in the virtual world of the sleeping brain.

**Fig. 1.**
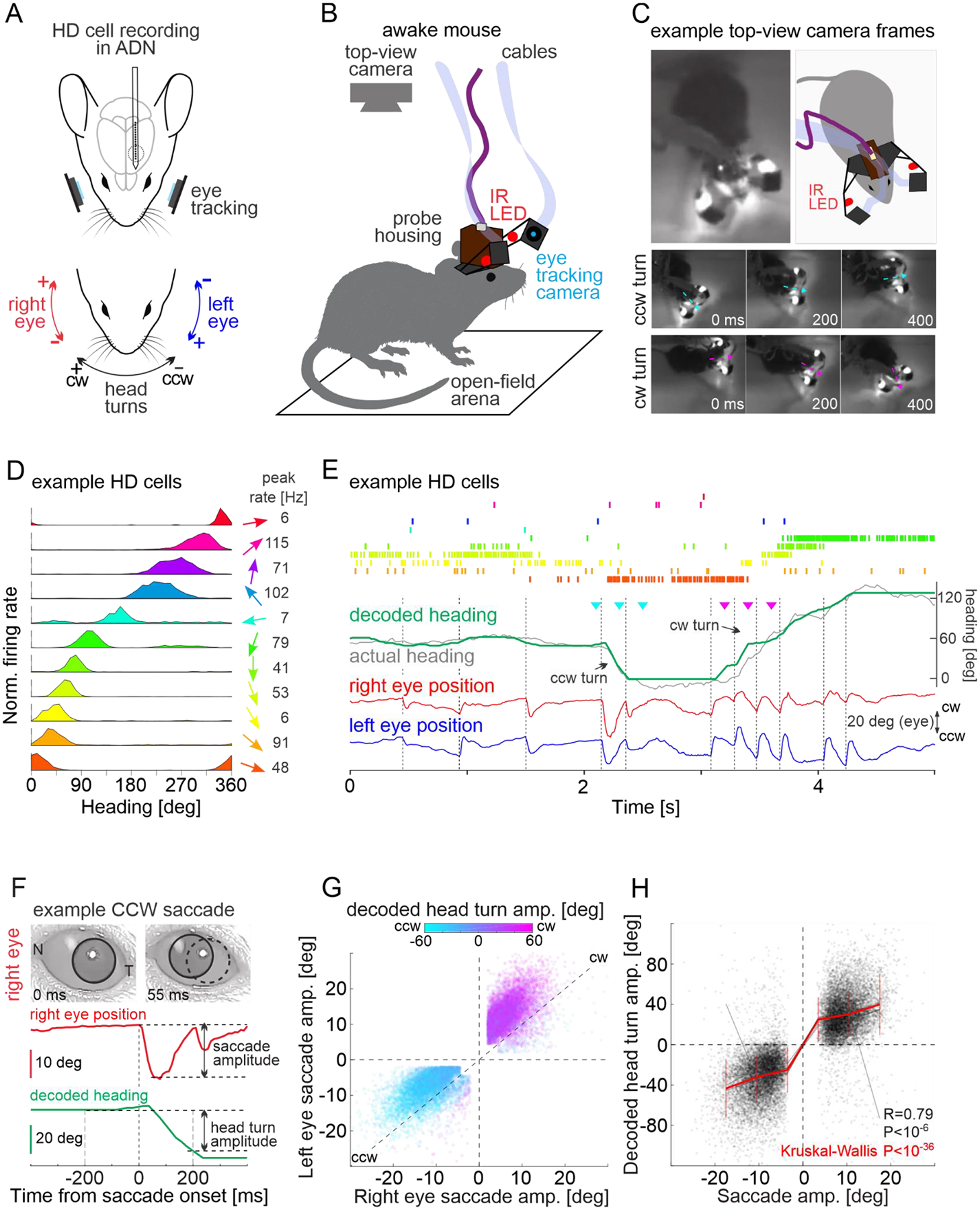
Saccade direction predicts internal representation of head turns. (**A**) Schematic of experimental configuration illustrating a chronic electrophysiological recording from the anterodorsal nucleus of the thalamus (ADN) while eye and head movements are monitored with cameras. (**B**) Schematic illustration of the open field arena with a mouse carrying head-mounted eye cameras and silicon probes. The heading of the animal is monitored with a top-view camera. (**C**) Top left: Example frame from the top-view camera. Top right: Schematic illustration of frame on the left. Middle: Example frames showing a counterclockwise (CCW) turn and, bottom, a clockwise (CW) turn. Arrows indicate the heading of the animal in each frame. (**D**) The tuning curves of eleven example head direction (HD) cells recorded from the ADN of an awake mouse. The arrow on the right indicates the preferred head direction of each HD cell. Peak firing rates for HD cells are shown on the right. (**E**) Top: Raster plot of the firing of the eleven example HD cells shown in (D). Middle traces: Actual heading of the animal (gray) and heading decoded from the population activity of HD cells (green). Triangles mark the timing of example frames for CCW turn (cyan) and CW turn (magenta) shown in (C). Bottom traces: Horizontal position of the two eyes (red and blue). The vertical dotted lines indicate the onset of saccades. (**F**) Top: Two snapshots of the right eye taken before and after a CCW saccade. The pupil is delineated with a black circle. A dotted circle in the right image labels the pupil’s original position (N: nasal commissure; T: temporal commissure). The red trace illustrates the right eye’s horizontal position in time. Saccade amplitude was defined as the change in the horizontal eye position upon a saccade. The green trace illustrates the decoded heading. The head turn amplitude is the change in the decoded heading between 200ms before and 200ms after saccade onset. Note the CCW shift in decoded heading concomitant with the CCW saccade. (**G**) Summary scatter plot of the amplitude of conjugated saccades during the exploration in the open field arena (n=13778 from 6 mice). The amplitude and sign of the decoded head turn during the saccade is color coded. Note that the direction of the saccades matches the direction of the decoded head turn. In this and the rest of the figures the upper right and lower left quadrants of the scatter plots represent CW and CCW movements, respectively. (**H**) Correlation between the amplitude of conjugated saccades (averaged over both eyes) with the decoded head turn amplitude. Saccade amplitude predicted the amplitude of decoded head turns for each individual mouse (gray lines) as well as for all mice (red line, vertical lines for standard deviation).

**Fig. 2.**
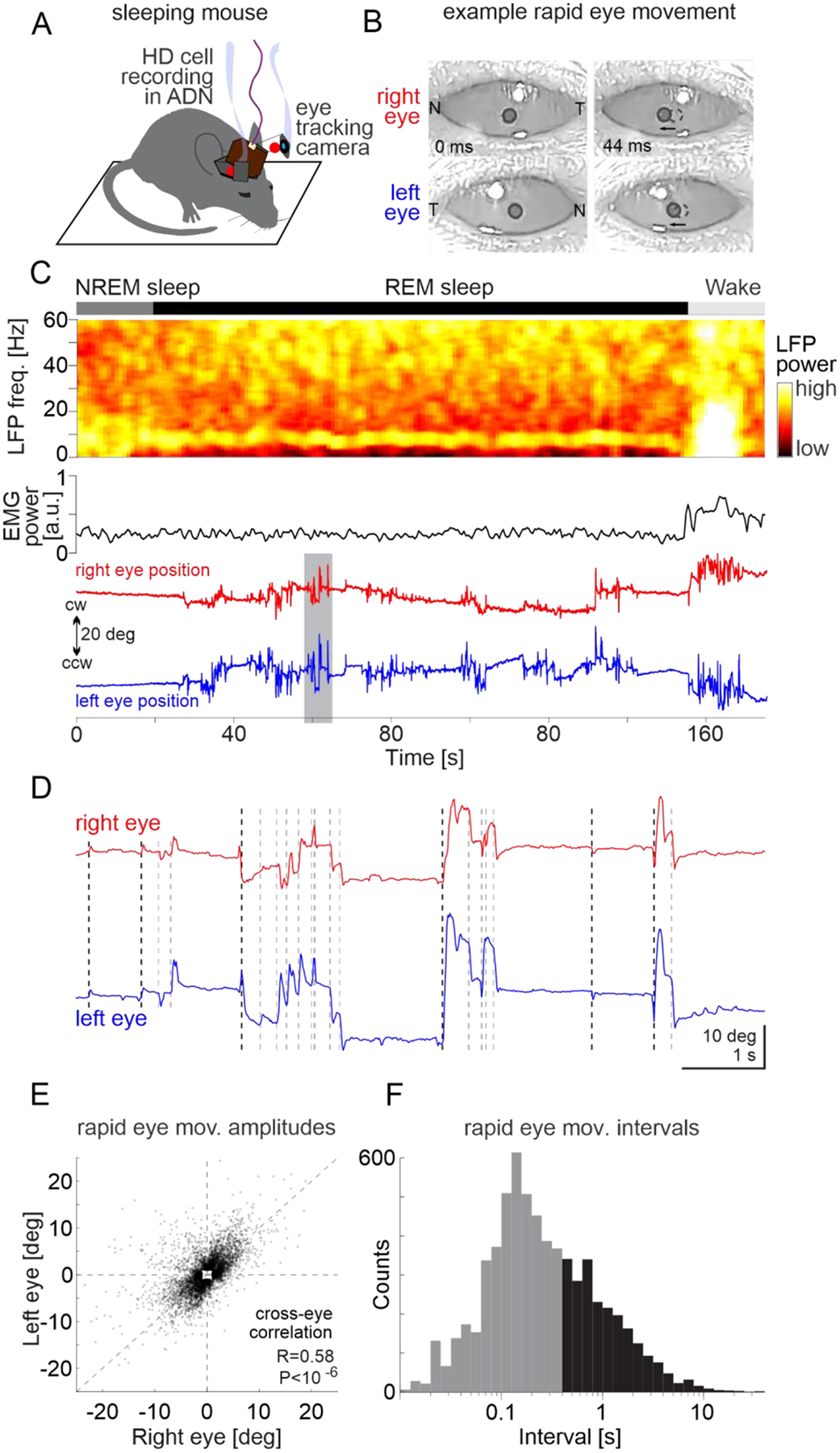
Rapid eye movements during REM sleep (**A**) Schematic of experimental configuration illustrating a chronic electrophysiological recording from the anterodorsal nucleus of the thalamus (ADN) while rapid eye movements are monitored with cameras in a sleeping mouse. (**B**) Snapshots of the right (top) and left eye (bottom) before (left) and after (right) a CCW rapid eye movement. The pupil is delineated with a black circle. A dotted circle in the right image labels the pupil’s original position (N: nasal commissure; T: temporal commissure). Note that both eyes move CCW. (**C**) Top: Example spectrogram of LFP recorded in the ADN during non-REM sleep (NREM), REM sleep and wakefulness (Wake). Middle: EMG power. Bottom: Horizontal position of the two eyes (red and blue). Note the increase in eye movements at the onset of REM sleep. (**D**) The shaded time interval in (C) shown on an expanded time scale. Note that for most rapid eye movements both eyes moved to the same direction. Dotted black and gray vertical lines indicate the onset of leading (not preceded by a rapid eye movement for at least 400 ms) and follower rapid eye movements. (**E**) Scatter plots of the amplitude of right versus left rapid eye movements during REM sleep for all mice (n=6689 from 6 mice). Note that most data points are in the lower-left or upper-right quadrants, indicating CCW and CW movements of both eyes. (**F**) Distribution of intervals between rapid eye movements during REM sleep for all mice (n = 6689 from 6 mice). Leading eye movements are in black and followers in gray.

**Fig. 3.**
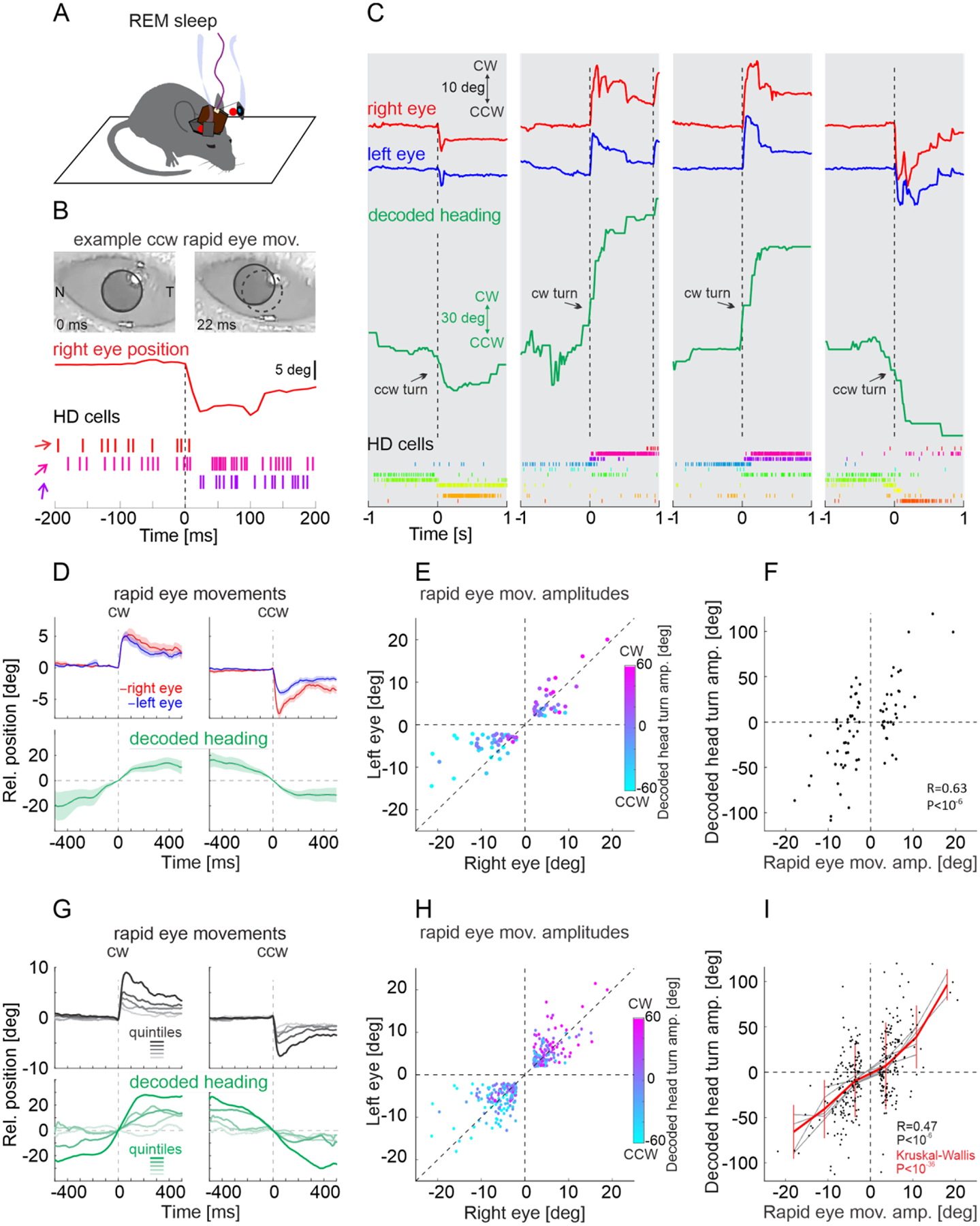
Leading rapid eye movements predict decoded head turns during REM sleep (**A**) Top: Schematic of experimental configuration (same as Fig. 2A) **(B-F)** Same mouse as in Fig. 1C–E. (**B**) Top: Two snapshots of the right eye during REM sleep taken before and after a CCW rapid eye movement. The red trace illustrates the right eye’s horizontal position in time. Bottom: Raster plot of the firing of three example HD cells (out of the eleven HD cells illustrated in Fig. 1D; same color code). Note the CCW shift in heading representation concomitant with the CCW rapid eye movement. (**C**) Four example episodes illustrating a concomitant shift in decoded heading and eye position during REM sleep. Top traces: Horizontal position of the two eyes (red and blue) and decoded heading (green). The vertical dotted lines indicate the onset of leading rapid eye movements. Bottom: Raster plot of the firing of the eleven example HD cells (same as in Fig. 1D). (**D**) Top: Average position of right (red) and left (blue) eye for CW (left) and CCW (right) leading eye movements. Bottom: Average decoded heading (green). Shaded areas are standard error of the mean (average of 34 traces for CW and 47 traces for CCW leading eye movements: same mouse as in B and C). (**E**) Scatter plot of the amplitude of leading right versus left eye movements during REM sleep. The amplitude and sign of the decoded head turns during the eye movements is color coded. Note the good match between the direction of the rapid eye movements and the direction of the decoded head turn (same mouse as in B and C). (**F**) Correlation between the amplitude of leading eye movements during REM sleep (averaged over both eyes) and the decoded head turn amplitude (same mouse as in B and C). (**G**) Top: Mean traces of horizontal eye position averaged across both eyes for CW (left) and CCW (right) leading eye movements (n = 6 mice). The dataset was separated into quintiles based on the amplitude. Bottom: Mean traces of the decoded heading in each quantile based on the amplitude of leading rapid eye movements. Note that leading rapid eye movements with larger amplitude coincide with larger decoded head turns. (**H**) Summary scatter plot of the amplitude of leading right versus left eye movements during REM sleep (n = 330 from 6 mice). The amplitude and sign of the decoded head turns during the eye movements is color coded. (**I**) Correlation between the amplitude of leading eye movements during REM sleep and the decoded head turn amplitude (n = 330 from 6 mice). Leading rapid eye movements predicted the direction and amplitude of decoded head turns in each individual mouse (gray lines) as well as for all mice (red line, vertical lines for standard deviation).

**Fig. 4.**
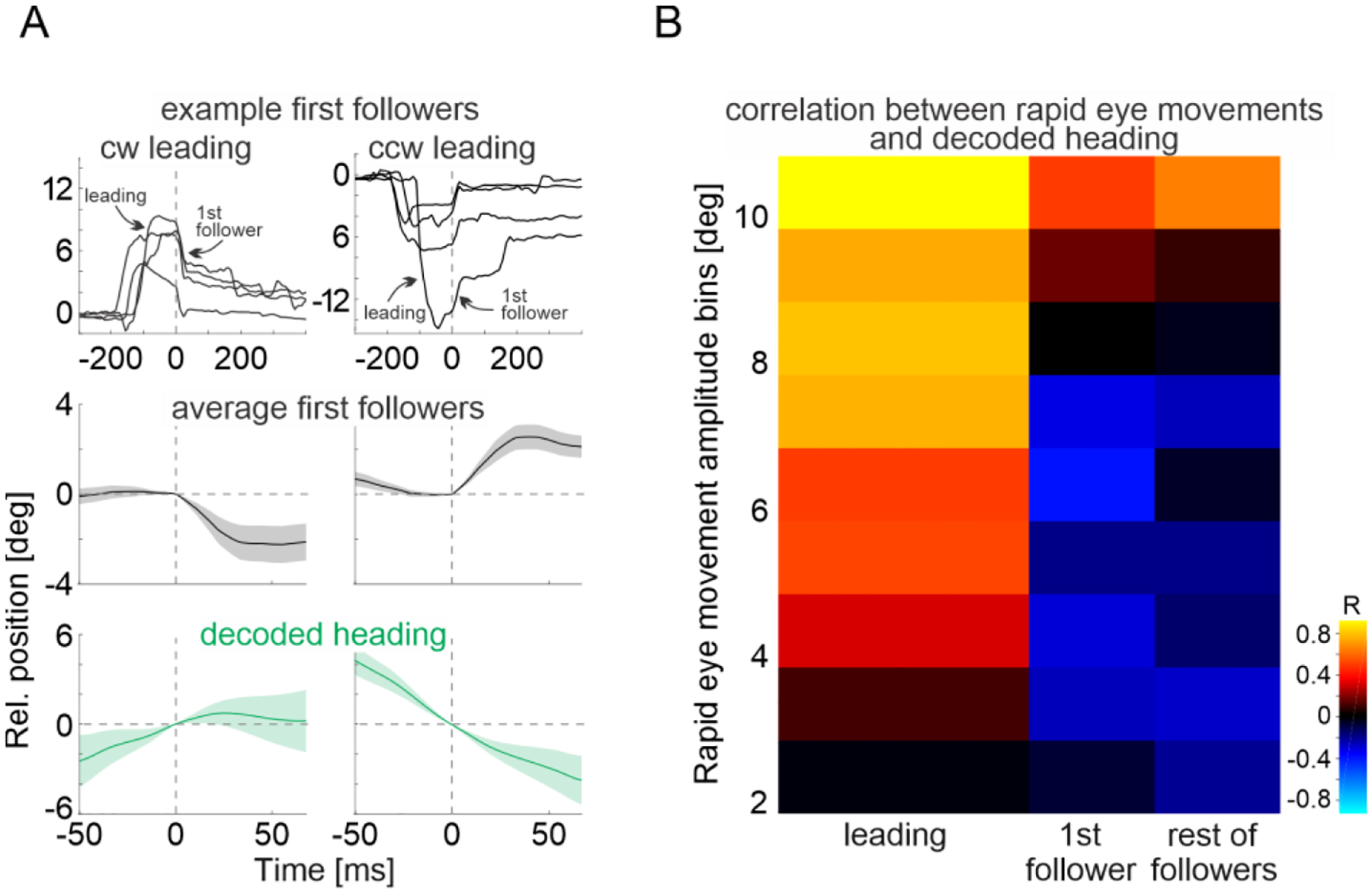
Small-amplitude follower eye movements represents recentering eye movements (**A**) Top: Example traces of horizontal eye position (averaged across both eyes) aligned to the onset of first follower rapid eye movement after a CW (left) and CCW (right) leading eye movement. Middle: Average horizontal eye position (mean of both eyes) for first follower eye movements after CW (left) and CCW (right) leading eye movement (average over 132X traces for CW and 107 traces for CCW from 6 mice). Bottom: Average traces of concomitantly decoded heading. Note that, on average, first followers occur in the opposite direction as compared to the preceding leading eye movement and to the decoded head turn. Shaded area represents standard error of the mean. The time scale of the top panels covers a larger interval to include the preceding leading eye movement. (**B**) Correlation (heatmap) between the decoded head turn amplitude and amplitude of rapid eye movement (in bins; averaged for both eyes; y-axis). First columns from left: Leading eye movements (n = 330 from 6 mice); second column: first followers (n = 239 from 6 mice), third column: rest of the followers (n = 970 from 6 mice). Note the increased positive correlation with increasing eye movement amplitude.

We recorded from HD cells in the ADN of mice with extracellular linear probes while monitoring the movements of both eyes with head mounted cameras (Fig. 1A; Movies S1 and S2). Mice were free to explore an open field arena and their heading was monitored with a top view camera (Fig. 1B). Mice were allowed to fall asleep and their sleep phase was identified as REM or non-REM using standard electrophysiological parameters (animals spent 40%−52% of the session asleep and 10% of the sleeping period were identified as REM sleep; Table. S1; see methods). To determine whether, during REM sleep, rapid eye movements are coordinated with the internal representation of heading, we proceeded through three steps: First, we established, in awake mice, the relationship between saccade-like eye movements and the internal representation of heading of the animal as decoded from the activity of HD cells (Fig. 1). Second, we determined the properties of rapid eye movements in mice during REM sleep (Fig. 2) and, finally, we investigated the nature of the relationship between rapid eye movements in REM sleep and the internal representation of heading (Fig. 3 and 4).

We identified saccade-like eye movements in awake animals based on their fast dynamics (>400 deg/sec). During the exploration of the open field arena, saccade-like eye movements occurred mainly along the naso-temporal axis (Fig. S1A, B) and the vast majority (94.1) of these eye movements were conjugated i.e. both eyes moved in the same direction, either clockwise (CW) or counterclockwise (CCW) (R = 0.89, P < 10^−6^; Fig. S1C). Below we focus on conjugated saccade-like eye movements of at least 2 degrees in amplitude and refer to them simply as saccades. CW and CCW saccades were coupled to head turns in the same direction i.e., with CW and CCW head turns along the azimuth, respectively, consistent with previous reports (21, 22) (Fig. S1E, F). To determine the relationship between the direction of saccades and the internal representation of heading, we analyzed the population activity of HD cells recorded in the ADN while the animal explored the open field arena (Fig. 1C and Fig. S2A, B). We recorded between 30–72 HD cells/animal (n = 6 mice. Table. S1) defined as neurons whose activity was modulated by the heading of the animal along the azimuth (Fig. 1D; see methods). Using the heading of the animal and the simultaneously recorded HD cells, we trained an algorithm to report the heading solely based on the firing of HD cells (see methods). When tested on untrained periods of HD cell activity, the algorithm accurately decoded the heading of the mice as they explored their environment (Fig. 1E) with an error of only 10.2+3.5 degrees (n = 6 mice. Fig. S2C, D; see methods). How well does the direction of a saccade match the internal representation of a head turn? We quantified the internal representation of a head turn as the difference between the heading decoded 200 ms before and 200 ms after a saccade (Fig. 1F). The vast majority of saccades (95.2 ±1.3%, n = 6 mice) occurred in the same direction (CW or CCW) as the ongoing head turns decoded from HD cell activity (Fig. 1G, H). In awake animals, saccade direction and the internal representation of head turns are thus tightly coupled.

Is this relationship maintained during REM sleep? To monitor rapid eye movements during REM sleep we took advantage of the fact that mice do not always close their eyes while asleep (Fig. 2A, B; Movie. S2). We quantified the direction and amplitude of rapid eye movements in the sleeping animal using the head mounted cameras (23, 24). During REM sleep (Fig. 2C; see methods), rapid eye movement occurred along the naso-temporal axis (Fig. S3A, B; monitored over a total of 68 min of REM sleep in 6 mice) and the direction of these movements, CW or CCW, was well correlated across both eyes (R = 0.58, P < 10^−6^; Fig. 2C–E), thus similar to saccades observed in awake animals (Fig. S3C), albeit with smaller amplitudes (average amplitude: rapid eye movements: 5.1 ± 2.5 deg; saccades 9.7 ± 3.5 deg) and at higher frequency (median interval: rapid eye movements: 267 ms; saccades: 521 ms; Fig. 2F and Fig. S1D).

We decoded the internal representation of heading during REM sleep i.e., the virtual heading, by applying the algorithm described above. Virtual heading was decoded from the activity of the same set of HD cells used to train the algorithm, this time, however recorded during REM sleep. Changes in virtual heading i.e., virtual turns, occurred with an angular velocity that was similar to that decoded during actual turns in awake mice exploring the arena (Fig. S4A, B), consistent with previous results (19). Furthermore, during REM sleep, HD cells maintained a similar correlational structure as that observed during wakefulness: HD cells that either fired or did not fire together in wakefulness exhibited the same pattern during REM sleep (Fig. S4C, D). To test the relationship between rapid eye movements and virtual heading, we first focused our analysis on rapid eye movements not preceded by any eye movement for at least 400 ms (Fig. 2F) and in which both eyes moved by at least two degrees in the same direction (i.e. conjugated rapid eye movements; see methods). This allowed us to have a sufficiently long baseline to detect potential changes in virtual heading specifically associated with the selected conjugated rapid eye movements. We refer to these rapid eye movements as “leading” eye movements. The direction of leading rapid eye movements matched the direction of simultaneously recorded virtual turns (Fig. 3A–C). A CCW leading rapid eye movement, for example, occurred as the ensemble activity of HD cells shifted CCW (Fig. 3B) and vice versa (Fig. 3C). Overall, during REM sleep, the direction of leading rapid eye movements predicted the direction of the changes in virtual heading, both in each individual mouse (Fig. 3D–F, Fig. S5) as well as in the population (Fig. 3G, H). Furthermore, not only the direction of the leading eye movements, but also their amplitude provided information about the ongoing internal representation of heading: the larger the amplitude of the leading rapid eye movement the larger the angle of the simultaneously recorded virtual turn (Fig. 3G–I).

What is the relationship between rapid eye movements that follow leading eye movements and the virtual heading of the animal? We refer to these eye movements as “followers”, i.e., occurring less than 400 ms after another rapid eye movement. The above results (Fig. 3D and G, top panels) show that the average position of the eye following a leading eye movement progressively returns to the original position occupied before the leading movement, as if recentering the eye (400 ms after a leading eye movement the eye returned to 34.1±3.3% (mean ± s.e.m.) relative to its peak position after the leading eye movement). In contrast, the simultaneously decoded virtual heading did not (Fig. 3D and G bottom panels). If the recentering of the eye is mediated by followers, the direction of these rapid eye movements should be opposite relative to that of leading eye movements and to the ongoing changes in virtual heading. Indeed, the direction of followers occurring immediately after a leading eye movement (1^st^ followers) were, on average, opposite to the direction of the preceding leading eye movement (Fig. 4A and Fig. S6A) and thus opposite to the direction of the ongoing head turns (Fig. 4A,B and Fig. S6B,C). Overall, the direction of followers was opposite to the direction of changes in virtual heading, but for the small fraction of followers (4.3 %) with the largest amplitudes (>10 degrees; Fig. 4B and Fig. S6D,E) that, like leading eye movement, matched the directions of virtual head turns.

Taken together, these data demonstrate a tight relationship between rapid eye movements and the internal representation of heading of the animal during REM sleep (Fig. 4B). Not only does the direction of leading rapid eye movements predict the direction of change in virtual heading, but their amplitude predicts the magnitude of the change. Follower eye movements, on the other hand, recenter the eye. Thus, our results demonstrate that rapid eye movements provide a readout of the internal representation of heading in the sleeping brain. The coordination between rapid eye movements during REM sleep and the head direction system suggests that shifts in virtual heading are part of a globally orchestrated representation of “virtual navigation” by the sleeping brains rather than the result of some uncorrelated random walk of the head direction system (20).

How do eye movements occurring during REM sleep map onto eye movements observed during wakefulness? Leading rapid eye movements may correspond to saccades because both match the direction of the ongoing head turn, virtual or real (21, 22, 25). Follower eye movements, however, appear unlike any eye movement in the awake animal. In the awake mouse, the recentering of the eye following a saccade is mediated by image stabilizing reflexes, the vestibular and optokinetic reflexes, that are engaged by the ongoing head-turn (21, 22, 25). In the sleeping, motionless animal, however, the sensory periphery that triggers these reflexes is not engaged. It is conceivable that recentering eye movements during REM sleep may still be triggered by activity in the vestibular nuclei. Such activity, while independent of the sensory periphery, could be part of the globally orchestrated virtual navigation mentioned above.

Whether rapid eye movements in REM sleep reveal the subjects’ direction of gaze in the imagery of dreams has been debated since the discovery of this sleep phase and its association with vivid dreams (7–13). Because of the lack of objective measures assessing the content of dreams, initial studies have led to conflicting results, leading to the conclusion that rapid eye movements are likely uncorrelated with the cognitive activity of the sleeping brain (12, 13). More recently, studies performed on human patients or animal models that partially enact their dreams because of reduced muscle atonia during REM sleep have led to a reevaluation of the original hypothesis (26, 27). In these studies, at least some rapid eye movements appeared coordinated with the direction of the behavior enacted during REM sleep. However, the extent to which the coordination between eye and body movement is a result of the pathological or experimentally reduced atonia still remains unclear.

In conclusion, by harnessing the head direction system of the rodent and the correlation between orienting head and eye movements in awake animals, we have established a clear relationship between changes in virtual heading and rapid eye movements during REM sleep. Thus, our results indicate that rapid eye movements provide an external readout of an internal cognitive process occurring during REM sleep, namely the change in virtual heading. Our results further suggest the existence of a globally coordinated activity among distinct systems in the sleeping brain during REM sleep, a coordination that may underlie the realistic and vivid experience of dreams. Understanding the neurophysiological mechanisms of this coordination will give us insight into the organization of the brain’s generative model of the world (28, 29).

## Supplementary Material

Video 1 Awake

Video 2 REM sleep

Supplementary Materials

## Data Availability

All data and analyses necessary to understand and assess the conclusions of the manuscript are presented in the main text and in the supplementary materials. Data and coded will be publicly available at Dryad (doi:10.7272/Q6P26WDC).
