# Supplementary material for: A cognitive process occurring during sleep revealed by rapid eye movements
Source: Science. Author manuscript; Available in PMC 2023 Feb 16. (PMC9933572; doi:10.1126/science.abp8852)
Supplement: Supplementary Materials [file NIHMS1849803-supplement-Supplementary_Materials.pdf]

Science
AAAS

# Supplementary Materials for

## Internal representation of heading during REM Sleep revealed by rapid eye movements

Yuta Senzai and Massimo Scanziani

Correspondence to: yuta.senzai@gmail.com; massimo@ucsf.edu

**This PDF file includes:**

Materials and Methods
Figs. S1 to S6
Table. S1
Captions for Movies S1 to S2

**Other Supplementary Materials for this manuscript include the following:**

Movies S1 to S2

## Materials and Methods

Animal

All experimental procedures were conducted in accordance with the regulations of the Institutional Animal Care and Use Committee (IACUC, AN179056) of the University of California, San Francisco. All mice were housed on a reversed cycle (light/dark cycle 12/12 h) with free access to food. Data were collected from male or female C57BL/6J mice (Jackson Laboratory #000664). At the start of the experiments, all mice were between 2.5 and 4 months old.

Surgery and electrode implantation

Mice were implanted under 2% isoflurane anesthesia. The procedure was performed in two steps: First, implantation of the headplate, second implantation of the silicon probe. The headplate base, integrated with infrared LEDs and 3D printed holders for eye tracking cameras (see below), was placed on the skull by dental cement (Unifast LC, GC America; OptiBond Universal, Kerr Dental). The camera holders were positioned such that each camera faced one eye. For this, the image plane of the cameras had to be perpendicular to an axis of +60° and -60° along the azimuth (relative to the antero-posterior axis of the mouse) and 30° elevation (relative to the horizontal plane defined as the plane that goes through bregma and lambda with 0 degrees roll; based on Sakatani and Isa, 2007 (*30*)). After a minimum post-op recovery of five days in their home cage, mice were habituated to freely explore the open field arena (see below) with their head attached to the cables for electrophysiological recordings and eye-tracking cameras. Following habituation to the experimental setup, mice underwent the second step, namely the implantation of the silicon probe. A two-shank silicon probe (Cambridge NeuroTech H4 64 or Diagnostic Biochips P64-4, 250 μm inter-shank distance, 32 channels per shank, 25 μm interchannel pitch) was mounted on a movable microdrive to record from the anterodorsal nucleus of the thalamus (ADN). Each of the two shanks of the probe were inserted, through a craniotomy, at the following coordinates, relative to Bregma: anteroposterior: -0.7 mm, mediolateral: +0.7 and +0.95 mm. The probe was initially lowered to 0.8-1.0 mm below the brain surface. After recovery from isoflurane anesthesia, the probe was lowered to the ADN by moving the microdrive over one to three days. A ground electrode (0.005" diameter stainless wire, A-M systems) was inserted in the cerebellum. The experiments were performed after waiting a minimum of 12 hours since the last probe depth adjustment.

Extracellular electrophysiological recordings

We recorded from the ADN of mice, both in their home cage as well as while exploring the open field arena for food, for 60-120 min sessions. The entire recording amounted to a total of 7 to 16 hours per mouse. Electrophysiological data were acquired using an Intan RHD2000 system (Intan Technologies LLC) band passed filtered between 0.1 Hz and 7.5 kHz and digitized at 20 kHz. Spike sorting was performed semi-automatically, using Kilosort 2.0 (*31*). This was followed by manual adjustment of the waveform clusters using the software Phy, which was performed blinded to other data. For the local field potential (LFP), the wide-band signal was down-sampled to 1.25 kHz.

Eye tracking

We adopted the head-mounted eye tracking system described in Meyer et al. 2018 (*32*). Light-weight camera modules (Arducam, with 5MP Omnivision OV5647 sensor) were used for eye tracking, one for each eye. Each camera was inserted into a 3D printed camera holder integrated in the head plate. Each eye was illuminated by an infrared LED (VSMB2943GX01, Vishay), also integrated in the head plate. Videos from the head mounted cameras were acquired at a frame rate of 90 Hz using single-board computers (Raspberry Pi 3 model B, Raspberry Pi Foundation), one for each camera, and controlled using a software described in Meyer et al. 2018 (*32*) and reposed in their github.

Open field arena

The rectangular open field arena (50 × 28 cm) was surrounded by 25 cm high walls displaying salient visual cues. The arena base and the walls were made from white plastic. The arena was illuminated by visible room lights as well as by infrared LED lights for video acquisition. The video (50 Hz sampling rate) was captured by a CMOS camera (Basler acA1300-200um) placed above the arena with infrared pass filter (Hoya) in front of the lens. The heading of the animal was detected based on the vector connecting the neck to the nose of the mouse. The extraction of the coordinates of the nose and the neck was performed by DeepLabCut (*33*). The heading was up-sampled to 100Hz by linear interpolation and smoothing (running average with a 210 ms window size).

Histological processing

For anatomical analysis, mice were perfused transcardially with phosphate buffered saline (PBS) and then with 4% paraformaldehyde (PFA) in PBS. Brains were extracted from the skulls, post-fixed in 4% PFA overnight at 4°C, and subsequently cut with a vibratome to 80-100 μm thick sequential coronal sections. Slices were collected and mounted in ProLong Gold (Life Technologies) or Vectashield mounting medium containing DAPI (Vector Laboratories H1500). Bright-field and fluorescence images were acquired using a Nikon Ti Inverted Microscope equipped with a Nikon DS-Qi2 monochrome camera (UCSF Nikon Imaging Center, 6D). The probe tracks were detected based on the gliosis formed around the probe.

Brain state scoring

We identified wakefulness, non-REM sleep and REM sleep using the approach described in Watson (2016) (*34*). Three metrics were extracted from the LFP data: power in the low-frequency band (<20 Hz), theta-ratio and electromyogram (EMG). To obtain the power in the low-frequency band, characteristically high in non-REM sleep, we first constructed spectrograms with a 1 s sliding 10 s-window Fast Fourier Transform (FFT) of the LFP at log-spaced frequencies between 1 and 100 Hz. We then performed principal components analysis (PCA) on the z-transformed (1-100 Hz) spectrogram and used the first PC as the power in the low-frequency band. The first PC mainly accounts for the variance in the spectrogram resulting from the alternation between "synchronous/low-frequency" (non-REM sleep) and "asynchronous/high-frequency" (Wakefulness/ REM sleep) states (*34*). The theta-ratio was calculated as the ratio of 5-10 Hz power to 2-16 Hz power. EMG was extracted by detecting the zero time-lag correlation coefficients between 300-600 Hz filtered signals recorded at all sites (*35*). Non-REM sleep periods were detected as intervals with high power in the low-frequency band. Among the remaining intervals, REM sleep periods were identified as those with weaker EMG values and stronger theta-ratio and the rest was wakefulness. Thresholds for each of the

three metrics were set at the trough of the bimodal distribution of each metric (see ref. *34*). After automated brain state scoring, all states were manually reviewed by the experimenter and minor corrections were made when discrepancies between automated scoring and user assessment occurred (*34*).

<u>HD cells</u>

The tuning of HD cells was computed as the ratio between histograms of spike count and total time spent in each direction in bins of 1 degree. The tuning of each HD cell was tested for non-uniformity with a Rayleigh test. Neurons with significant non-uniformity ($p<0.01$) were identified as HD cells. For the cross-correlation analysis of the firing of HD cell pairs (Fig. S4D), we included only HD cells with narrow-width tuning curves. We selected such HD cells based on the concentration parameter (comparable to the inverse of the variance) of the von Mises distribution, fitted to the HD cell tuning curve. Only HD cells with a concentration parameter larger than 2 were included in cross-correlation analysis.

<u>Bayesian decoding of heading</u>

Heading was decoded from the population activity of HD cells with a naive Bayesian decoding method using the toolbox described in Meyers (2013) (*36*). The firing rate of each HD cell was calculated over 10 ms bins and smoothed with a moving average filter (210 ms window). The data in the open field arena were split into two; the first half for training and the second half for testing. We used training data to calculate the mean firing rates of each HD cell in response to each heading. These mean rates were used as parameters to calculate a Poisson distribution for each HD cell in response to each heading. Based on these Poisson distributions, we calculated, for each HD cell, the likelihood of heading given the firing rate. These probabilities were multiplied across all HD cells to give an overall likelihood value for each heading. The heading with the highest likelihood value was chosen as the decoded heading. The decoder was applied to the testing data to evaluate the decoding accuracy.

<u>Eye tracking and angular eye position</u>

Because the eyelids may cover the top and bottom segments of the pupil, for each eye, we tracked the most nasal and temporal points along the pupil's edges using DeepLabCut (*33*). The nasal and temporal eye corners were also tracked with DeepLabCut (*33*). 50-100 randomly selected frames were labeled manually to train the deep network. The pupil center was defined as the middle point between the temporal and the nasal points of the pupil. Angular eye positions were computed based on a modification of the method described in Meyer et al. (2018, 2020) (*21, 32*). We converted pixels into metric units by using the length of the axis connecting the nasal and temporal eye corners (the horizontal eye axis; approximate length: 3.2 mm), averaged over the period that the animal spent in the open field arena. The center of the horizontal eye axis was assumed to be the point of tangency with a sphere whose radius connects the center of the eyeball to center of the pupil (estimated at 1.25 mm (Sakatani and Isa (2004) (*37*)). The angular position of the eye was computed using trigonometry. The horizontal angular eye position was defined as the orthogonal projection of the pupil center onto the horizontal eye axis.

<u>Detecting rapid eye movements and saccades in two dimensions</u>

We detected rapid eye movement (during REM sleep) and saccades (during exploration) by applying a speed threshold for eye movements in a two-dimensional plane defined by the naso-

temporal (horizontal) axis and the dorso-ventral (vertical) axis (Fig. S1A, B and Fig. S3A, B). Rapid eye movements were separately detected in each eye using a threshold of > 100 deg/s (i.e. > 1.1.deg/frame). The beginning and the end of each rapid eye movement, i.e. the duration, was defined as the first and the last time point, respectively, at which the velocity of the eye movement exceeded 50 deg/s. The amplitude and the direction of each rapid eye movement was defined by the vector connecting the original eye position to the furthest point the eye reached during the eye movement. Saccades were detected in the same way as rapid eye movements with different speed thresholds: 400 deg/s (4.4 deg/frame) for detection and 100 deg/s for duration. We used a higher velocity threshold to detect saccades as compared to that used to detect rapid eye movements. This higher threshold reduced the fraction of non-saccade gaze-stabilizing counter-rotations of the eye among the detected eye movements. Having established that most rapid eye movements and saccades occur along the horizontal axis (Fig. S1A, B and Fig. S3A, B), we used only the horizontal angular position of the eye for detecting rapid eye movements and saccades (see below).

Detecting horizontal rapid eye movements during REM sleep

The horizontal angular position of the eye (see section: Eye tracking and angular eye position, above) was used for detecting rapid eye movements during REM sleep. Rapid eye movements were first detected in each eye using a threshold of > 100 deg/s (> 1.1.deg/frame) along the horizontal eye axis. The duration or rapid eye movements was defined as the first and the last time point, respectively, at which the velocity of the eye movement exceeded 50 deg/s, along the horizontal eye axis. The amplitude of each rapid eye movement was defined as the change in the horizontal angular eye position within these two time points. If two consecutive eye movements in opposite directions were detected such that the last time point of the first movement preceded the first time point of the second movement by less than 50 ms, we considered only the eye movement with the largest amplitude. If a rapid eye movement was detected in one eye only, the amplitude of the eye movement in the other eye was defined as the change in the horizontal angular eye position occurring during the duration of the rapid eye movement detected in the former eye. We defined rapid eye movements as occurring simultaneously in both eyes when the duration of the rapid eye movement detected in each eye overlapped, in time, by more than 50%. The onset of simultaneous eye movement was defined as the beginning of the rapid eye movement with the largest amplitude. When rapid eye movements between both eyes overlapped in time by less than 50% (i.e. between 0 and <50%) we disregarded the rapid eye movement with smaller amplitude and treated the event as if the rapid eye movement was detected in one eye only (see above). To avoid the spurious detection of rapid eye movements caused by unstable labeling of the pupil edges by the DeepLabCut, we removed from the analysis rapid eye movements that coincided with > 0.02mm displacement of the temporal point relative to the nasal point along the pupil's edge across adjacent video frames. Rapid eye movements were defined as conjugated when both eyes moved in the same direction (i.e. clockwise or counterclockwise) by at least two degrees.

Detecting horizontal saccades during exploration

The detection of saccades during wakefulness was performed in the same way as the detection of rapid eye movements during REM sleep, except for some parameters because of the difference in the dynamics of the two types of eye movements. Saccades were detected in each eye using a threshold of > 400 deg/s (i.e. 4.4 deg/frame). The beginning and the end of each

saccade was defined as the first and the last time point, respectively, at which the velocity of the eye movement exceeded 100 deg/s. The amplitude of each saccade was defined as the change in the horizontal angular eye position within these two time points. If two saccades in opposite directions were detected such that the last time point of the first movement preceded the first time point of the second movement by less than 100 ms, we considered only the fastest eye movement. If a saccade was detected in one eye only, the amplitude of the eye movement in the other eye was defined as the change in the horizontal angular eye occurring during the saccade detected in the former eye. We defined saccades as occurring simultaneously in both eyes when the duration of the saccade detected in each eye overlapped, in time, by more than 50%. The onset of simultaneous eye movement was defined as the beginning of the saccade with the largest amplitude. When saccades between both eyes overlapped in time by less than 50% (i.e. between 0 and <50%) we disregarded the saccade with smaller amplitude and treated the event as if the saccade was detected in one eye only. To avoid the spurious detection of saccades caused by unstable labeling of the pupil edges by the DeepLabCut, we removed from the analysis saccades that coincided with >0.02 mm displacement of the temporal point relative to the to the nasal point along the pupil's edge across adjacent video frames. Saccades were defined as conjugated when both eyes moved in the same direction (i.e. clockwise or counterclockwise) by at least two degrees.

<u>Quantification and statistical analysis</u>

All statistical analyses were performed with standard MATLAB (MathWorks) functions. No specific analysis was used to estimate minimal population samples but the number of animals, recorded cells, detected eye movements were larger or similar to those employed in previous works (*19, 21, 22*). Kruskal-Wallis one-way analysis of variance and Rayleigh test were used. Correlations were computed using Pearson's correlation coefficient. Unless stated otherwise, all values in the text are given as mean average ± standard deviation.

**References**


30. T. Sakatani, T. Isa, Quantitative analysis of spontaneous saccade-like rapid eye movements in C57BL/6 mice. *Neurosci. Res.* **58**, 324–331 (2007).

31. C. Stringer, M. Pachitariu, N. Steinmetz, C. B. Reddy, M. Carandini, K. D. Harris, Spontaneous behaviors drive multidimensional, brainwide activity. *Science*. **364**, 255 (2019).

32. A. F. Meyer, J. Poort, J. O'Keefe, M. Sahani, J. F. Linden, A Head-Mounted Camera System Integrates Detailed Behavioral Monitoring with Multichannel Electrophysiology in Freely Moving Mice. *Neuron*. **100**, 46–60.e7 (2018).

33. A. Mathis, P. Mamidanna, K. M. Cury, T. Abe, V. N. Murthy, M. W. Mathis, M. Bethge, DeepLabCut: markerless pose estimation of user-defined body parts with deep learning. *Nat. Neurosci.* **21**, 1281–1289 (2018).

34. B. O. Watson, D. Levenstein, J. P. Greene, J. N. Gelinas, G. Buzsáki, Network Homeostasis and State Dynamics of Neocortical Sleep. *Neuron*. **90**, 839–852 (2016).

35. E. W. Schomburg, A. Fernández-Ruiz, K. Mizuseki, A. Berényi, C. A. Anastassiou, C. Koch, G. Buzsáki, Theta phase segregation of input-specific gamma patterns in entorhinal-hippocampal networks. *Neuron*. **84**, 470–485 (2014).

36. E. M. Meyers, The neural decoding toolbox. *Front. Neuroinform.* **7**, 8 (2013).

37. T. Sakatani, T. Isa, PC-based high-speed video-oculography for measuring rapid eye movements in mice. *Neurosci. Res.* **49**, 123–131 (2004).

**Fig. S1**

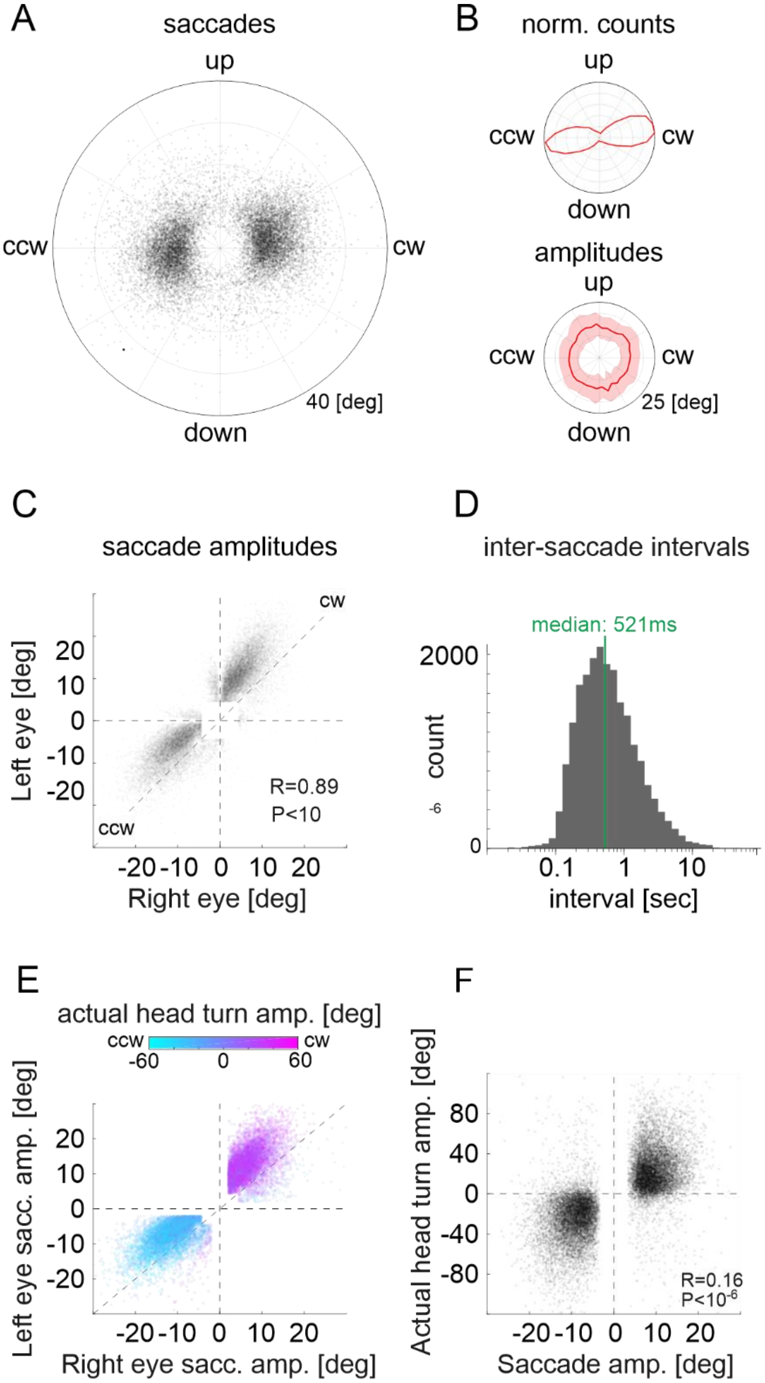
A
saccades
up
ccw
cw
40 [deg]
down
B
norm. counts
up
ccw
cw
down
amplitudes
up
ccw
cw
25 [deg]
down
C
saccade amplitudes
cw
ccw
R=0.89
P<10-6
Left eye [deg]
Right eye [deg]
D
inter-saccade intervals
median: 521ms
count
interval [sec]
E
actual head turn amp. [deg]
ccw
cw
-60
0
60
Left eye sacc. amp. [deg]
Right eye sacc. amp. [deg]
F
Actual head turn amp. [deg]
Saccade amp. [deg]
R=0.16
P<10-6

**Fig. S1. Conjugated saccadic eye movements in awake freely-moving mice.**

(**A**) Scatter polar plot of saccade directions and amplitudes during exploration in the open field arena. Data from both eyes (n = 13425 saccades). CCW and CW are nasal and temporal, respectively, for the right eye and vice versa for the left eye. Note that most saccades occur along the naso-temporal axis for all mice (n = 6).

(**B**) Top: Normalized counts of saccade occurrence in each binned direction. Bottom: Mean amplitude of saccade in each binned direction for all mice (n = 6). Red shaded area represents standard deviation.

(**C**) Scatter plot of the amplitude of right versus left saccades for all mice (n = 6). In this and the rest of the supplementary figures the upper right and lower left quadrants of the scatter plots represent CW and CCW movements, respectively.

(**D**) Distribution of the intervals between saccades for all mice (n = 6). Green vertical line shows the median of the distribution.

(**E**) Summary scatter plot of the amplitude of conjugated saccades during the exploration in the open field arena (n = 6 mice). The amplitude and sign of the actual head turn (monitored with the top view camera) during the saccade is color coded.

(**F**) Correlation between the amplitude of conjugated saccades (averaged over both eyes) with the actual head turn amplitude.

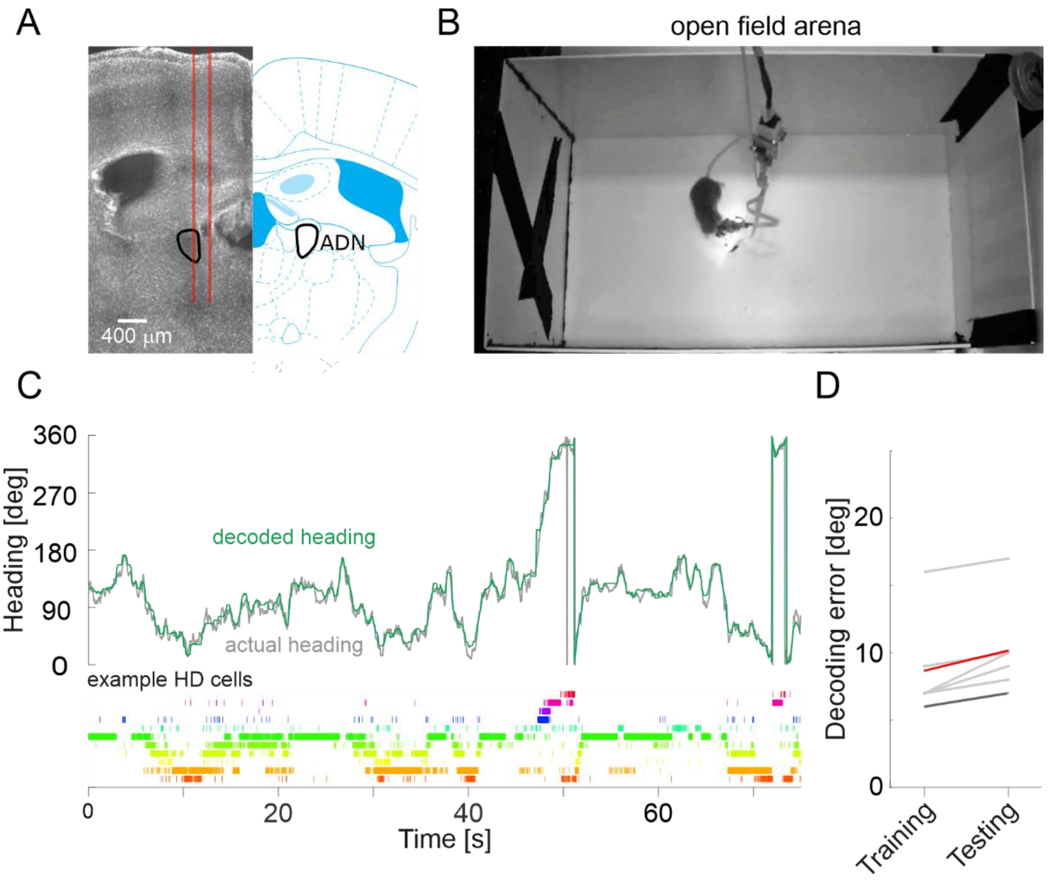


**Fig. S2. Decoding heading from HD cells in the ADN of awake mice.**

(**A**) Left: Micrograph of a DAPI-stained coronal section illustrating the probe tracks (red lines) targeted to the ADN (delineated in black). Right: Corresponding coronal plane from The Mouse Brain in Stereotaxic Coordinates by Franklin and Paxinos, for reference.

(**B**) Example frame from the top-view camera. The open field arena was surrounded by visual cues on the wall.

(**C**) Top: Actual (gray) and decoded heading (green) of an example period (75 s) of the mouse shown in Fig. 1C-E exploring the open field arena. Bottom: Raster plot of the firing of the eleven example HD cells shown in Fig. 1D (same color code).

(**D**) Median decoding error in the training and testing datasets for each individual mouse (gray), for the mouse shown in (C) (black) and for the mean of all mice (red).

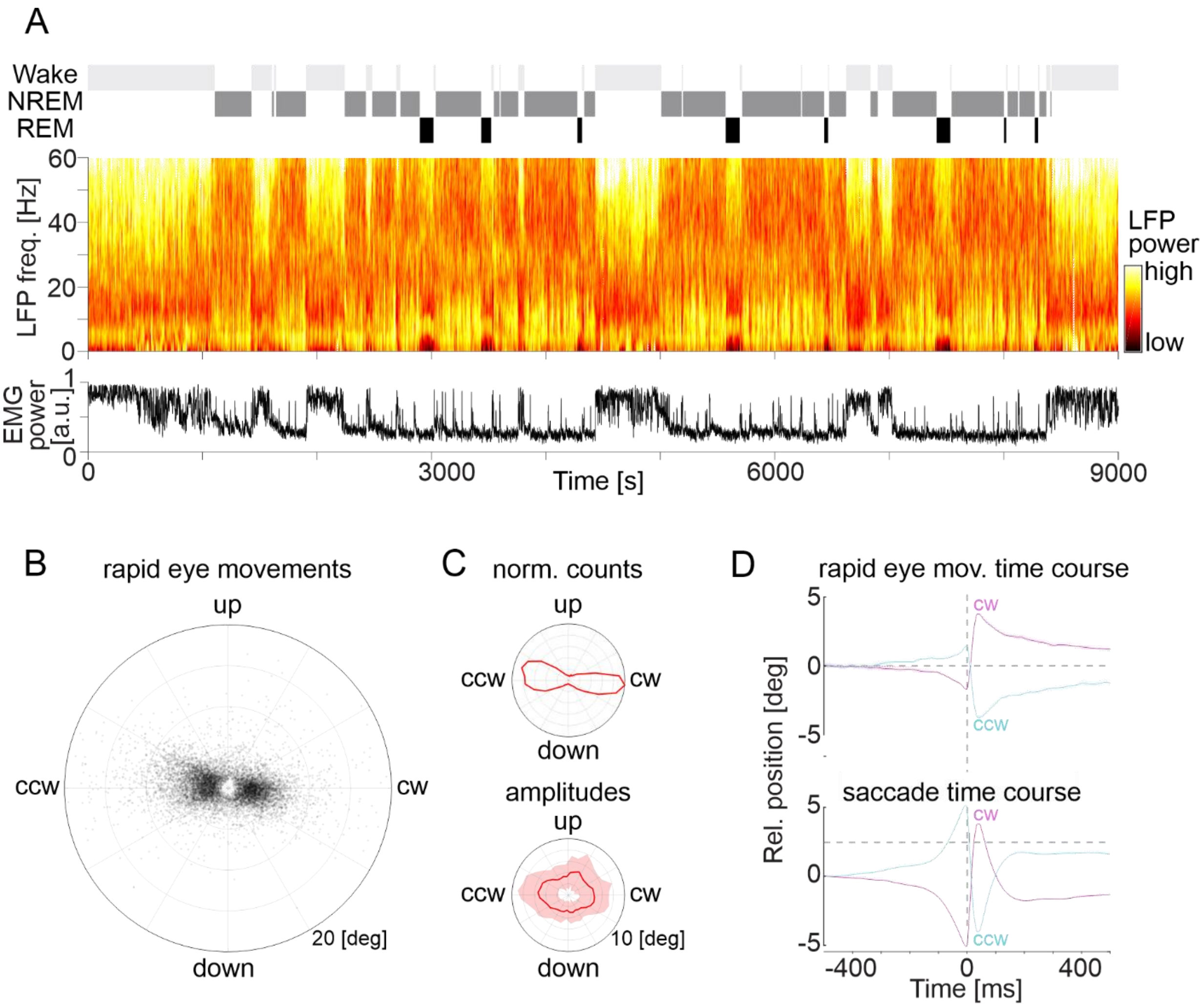


**Fig. S3. Rapid eye movements during REM sleep occur mostly along the naso-temporal axis.**

**(A)** Detection of REM sleep periods based on LFP and EMG power in an example period of 2.5 hours in the home cage. Top: state labels. Middle: LFP power spectrum. Bottom: EMG power.
(**B**) Scatter polar plot of rapid eye movement directions and amplitudes during REM sleep. Data from both eyes (n = 9050 rapid eye movements). CCW and CW are nasal and temporal, respectively, for the right eye and vice versa for the left eye. Note that most rapid eye movements occur along the naso-temporal axis for all mice (n = 6).
(**C**) Top: Normalized counts of rapid eye movement occurrence in each binned direction. Bottom: Mean amplitude of rapid eye movements in each binned direction for all mice (n = 6). Red shaded area represents standard deviation.
(**D**) Average time course of conjugated rapid eye movements during REM sleep (top) and of saccades in awake animals (bottom), for comparison. Magenta and cyan are CW and CCW eye movements, respectively. Shaded areas represent standard error of the mean. Note the larger average amplitude of saccades and the fact that the eyes return faster to their original position following saccades as compared to rapid eye movements. For this comparison, the same detection threshold (4 deg) was used for both rapid eye movements and saccades.

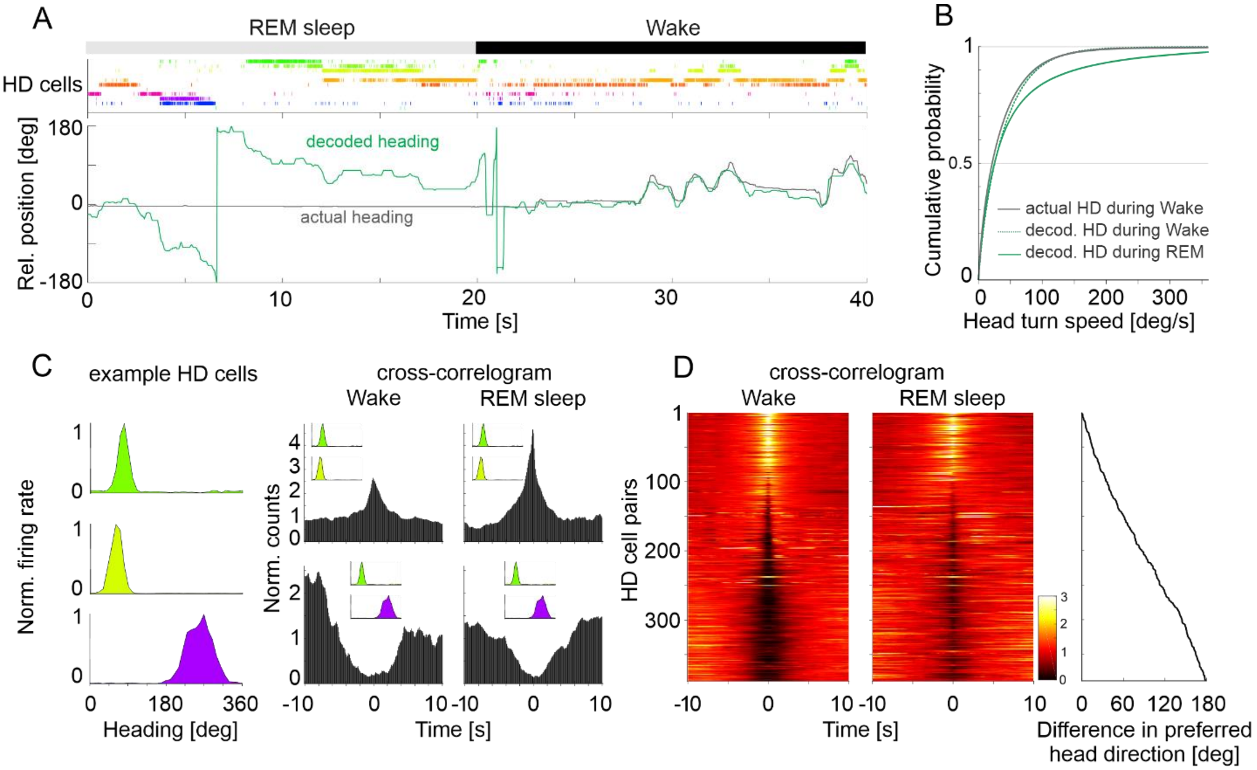


**Fig. S4. Similar activity of HD cells during wakefulness and REM sleep.**

**(A)** Top: Raster plot of the firing of the eleven example HD cells shown in Fig. 1D (same color code) during an example period of REM sleep (20 s) and immediately following wakefulness (Wake; 20 s). Bottom: Actual heading (gray) and decoded heading (green) of the same example period. Note that during REM sleep the actual heading is not changing, consistent with the immobility of the sleeping mouse.

**(B)** Cumulative distribution for the angular velocity of the actual heading during exploration (gray), decoded heading during exploration (dotted green), and decoded heading during REM sleep (continuous green).

**(C)** Left: The tuning curves of three example HD cells tuned to a similar direction (green and lime) or opposite direction (green and purple) (from Fig 1; same color code). Right: Cross-correlograms of the example HD cells shown on the left during wakefulness and REM sleep. Top: Cross-correlogram between the green and lime HD cells. Bottom: Cross-correlogram between the green and purple HD cells. Note that HD cells with similar preferred directions fire together both during wakefulness and REM sleep, while HD cells with opposite preferred directions do not fire together during wakefulness nor REM sleep.

**(D)** Left and middle panels: Cross-correlograms (heatmaps) of pairs of HD cells during wakefulness (left) and REM sleep (middle) from all mice (n = 6). The correlograms are ranked based on the difference between the preferred directions of the correlated HD cell pairs, with the smallest difference on top. The differences are plotted on the right panel. Note that the correlational structure of HD cell pairs during wakefulness is preserved during REM sleep. Only HD cells with narrow-width tuning curves were included in the analysis (see methods).

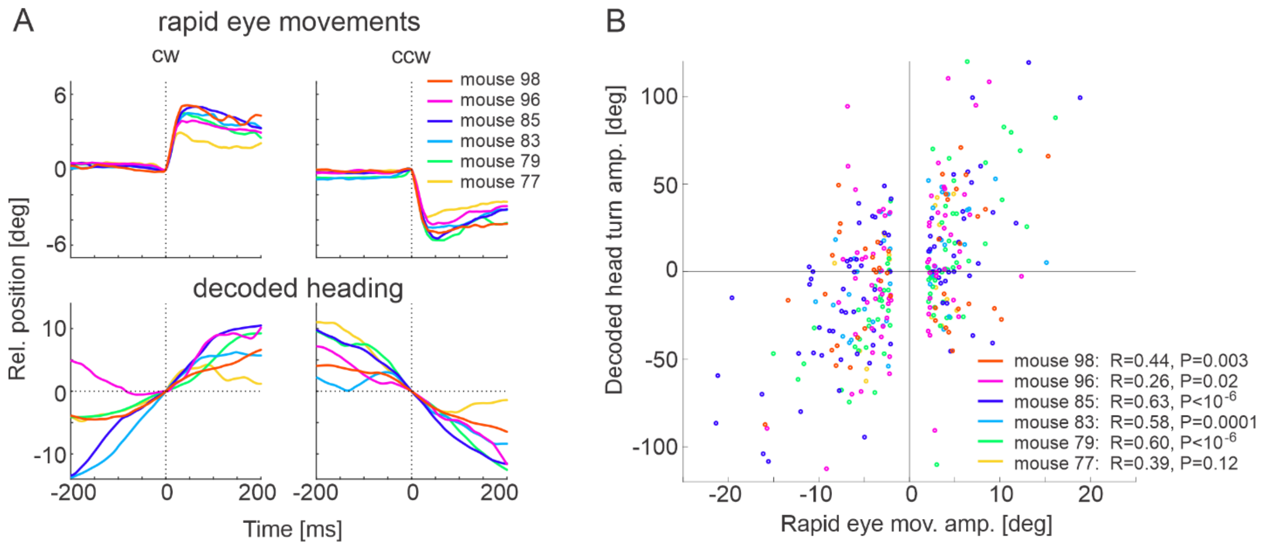


**Fig. S5. Leading rapid eye movements predict decoded head turns during REM sleep in each mouse**

**(A)** Top: Average eye position (averaged over both eyes) for CW (left) and CCW (right) leading eye movements. Bottom: Average decoded heading. Traces with different colors represent different mice.

**(B)** Correlation between the amplitude of leading eye movements during REM sleep and the decoded head turn amplitude, for each mouse.

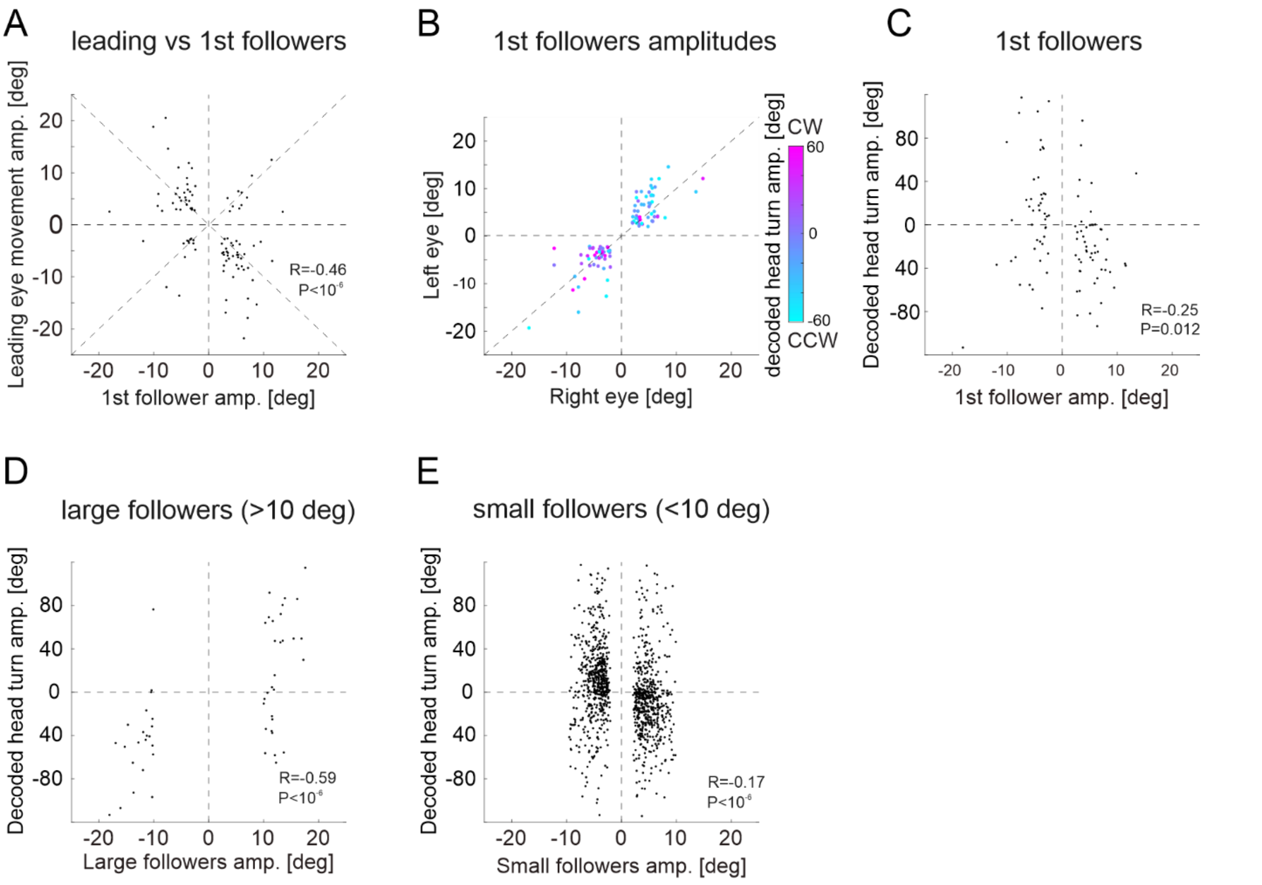


**Fig. S6. The direction of follower rapid eye movements is mainly opposite to that of decoded head turns during REM sleep.**

(**A**) Scatter plot of the amplitude of leading versus first follower rapid eye movements. Note that they occur mostly in opposite directions.

(**B**) Scatter plot of the amplitude of first followers of the right versus left eye during REM sleep (n = 6 mice). The amplitude and sign of the decoded head turns during the eye movements is color coded. Note that CW followers (upper right quadrant) occur mainly together with CCW decoded head-turns (cyan) while CCW followers (lower left quadrant) occur mainly together with CW decoded head turns (magenta).

(**C**) Scatter plot of the amplitude of first followers during REM sleep versus decoded head turn amplitude (n = 6 mice). Note that the direction of the first follower is mostly opposite to the direction of decoded head turns.

(**D**) Scatter plot of decoded head turn amplitudes versus follower rapid eye movements with amplitudes larger than 10 degrees during REM sleep. Note the good match between the direction of those large followers and the direction of the decoded head turns.

(**B**) Scatter plot of decoded head turn amplitudes versus follower rapid eye movements with amplitudes smaller than 10 degrees during REM sleep. Note that the directions of small followers were mostly opposite to the direction of decoded head turns.

| mouse ID | 77 | 79 | 83 | 85 | 96 | 98 |
|---|---|---|---|---|---|---|
| sex | M | M | M | M | F | F |
| Age @ implant [month] | 2.5 | 4 | 3.4 | 3.7 | 3.8 | 4 |
| Age @ recording [month] | 2.9 | 4.5 | 3.9 | 4 | 4.2 | 4.3 |
| Entire recording session [s] | 27582 | 34846 | 30055 | 25589 | 50082 | 56994 |
| Sleep duration [s] | 10902 | 15977 | 12958 | 11377 | 26140 | 29190 |
| REM sleep duration [s] | 1119 | 1788 | 1140 | 881 | 3250 | 3029 |
| Pupil detection during REM sleep [s]* | 232 | 810 | 833 | 819 | 1031 | 336 |
| Number of REM sleep bouts | 15 | 33 | 20 | 16 | 44 | 56 |
| Detected rapid eye movements | 208 | 1352 | 1413 | 1373 | 1740 | 603 |
| Total number of recorded neurons | 91 | 58 | 86 | 97 | 213 | 184 |
| Number of HD cells | 37 | 36 | 32 | 45 | 30 | 72 |
| HD decoding error (training) [deg] | 16 | 7 | 7 | 6 | 9 | 7 |
| HD decoding error (testing) [deg] | 17 | 10 | 8 | 7 | 10 | 9 |

*periods during which the eyes were open and the labeling of the pupil by DeepLabCut was stable (see methods).

**Table. S1. Summary of experimental parameters for each mouse.**

**Movie S1. Saccadic eye movements in a freely-moving mouse.**

Video taken by head mounted cameras (8.3 s played at a 0.28x) showing the eye movements of an awake, freely-moving mouse in the open field arena (left eye on the left; right eye on the right).

**Movie S2. Rapid eye movements during REM sleep.**

Same mouse as in Movie S1. Video taken by head mounted cameras (8.3 s played at a 0.28x) showing rapid eye movements during REM sleep.
